# Cortical waste clearance in normal and restricted sleep with potential runaway tau buildup in Alzheimer’s disease

**DOI:** 10.1038/s41598-022-15109-6

**Published:** 2022-08-12

**Authors:** Tahereh Tekieh, P. A. Robinson, Svetlana Postnova

**Affiliations:** 1 School of Physics, University of Sydney, Sydney, NSW 2006 Australia; 2 Center of Excellence for Integrative Brain Function, University of Sydney, Sydney, NSW 2006 Australia; 3 Woolcock Institute of Medical Research, University of Sydney, Sydney, NSW 2037 Australia; 4 Sydney Nano, University of Sydney, Sydney, NSW 2006 Australia; 5 Charles Perkins Center, University of Sydney, Sydney, NSW 2006 Australia

**Keywords:** Biophysics, Neuroscience, Physics

## Abstract

Accumulation of waste in cortical tissue and glymphatic waste clearance via extracellular voids partly drives the sleep-wake cycle and modeling has reproduced much of its dynamics. Here, new modeling incorporates higher void volume and clearance in sleep, multiple waste compounds, and clearance obstruction by waste. This model reproduces normal sleep-wake cycles, sleep deprivation effects, and performance decreases under chronic sleep restriction (CSR). Once fitted to calibration data, it successfully predicts dynamics in further experiments on sleep deprivation, intermittent CSR, and recovery after restricted sleep. The results imply a central role for waste products with lifetimes similar to tau protein. Strong tau buildup is predicted if pathologically enhanced production or impaired clearance occur, with runaway buildup above a critical threshold. Predicted tau accumulation has timescales consistent with the development of Alzheimer’s disease. The model unifies a wide sweep of phenomena, clarifying the role of glymphatic clearance and targets for interventions against waste buildup.

## Introduction

In recent years, biophysical models of arousal dynamics have been remarkably successful in explaining many features of normal sleep-wake cycles and the effects of acute sleep deprivation, as well as circadian dynamics, effects of stimulants, and circadian misalignment^1,2^. These models generally include two main drives: a circadian drive that is generated by the suprachiasmatic nucleus and oscillates with an approximately 24-h period that is normally entrained to the day–night light cycle—the “circadian clock”—and a homeostatic drive toward sleep that reflects the buildup of waste products in cortical tissue during waking hours, and their clearance during sleep^3–7^. Waste products are cleared from cortical tissue by the glymphatic system. This involves cerebrospinal fluid flowing from the subarachnoid space to periarterial spaces, then permeating a dense network of interstitial voids of several tens of nm transverse size that extend between cortical cells, before exiting via perivenous spaces, removing waste products in the process^7–12^. Various alternative suggestions have been made as to the nature of the flow of waste which mainly revolve around whether it is primarily advective, as just described, or whether waste diffuses through interstitial spaces to reach the perivascular ones without significant net advective flow, with diffusion possibly enhanced by pulsation of cerebrospinal fluid; likewise the relative roles of periarterial, perivenous, and interstitial spaces are all still controversial^5,7,8,11,13–17^. However, we model the above-described process here for definiteness and to provide a basis for future refinements.

Recently, it was found that the volume of interstitial spaces in mice increases by about 60% in slow wave sleep (SWS) relative to wake^8^, possibly driven by changes in norepinephrine concentration, which implies that both advection and diffusion will be enhanced. Parallel changes in perivascular volume do not appear to have been documented, but would have analogous effects if they exist. Simple scaling arguments from fluid mechanics (discussed further below) imply that a 60% increase in void cross section (void length should not change by a significant factor) should yield a factor of 2.56 ($=1.6^2$) enhancement in flux and hence in clearance rate for advection, and a factor of 1.6 for diffusion. Indeed, clearance rates of beta-amyloid in the sleeping mouse are around 2.5 times as large as in wake^8^. However, differential clearance between sleep and wake has yet to be incorporated in homeostatic modeling.

Modeling has succeeded in describing many aspects of the dynamics of normal arousal states and acute sleep deprivation over a few days by using a simple approximation of exponential clearance with a single time constant in the range of 18 to 60 h^1,2^. However, a vast spectrum of waste chemicals is produced by cortical cells, and these have very different observed clearance times. These include adenosine, prostaglandin D2, growth hormone-releasing hormone, interleukin-1, nitric oxide, and tumor necrosis factor, some of which promote non-REM sleep in various animal species^18,19^. Other chemicals such as amyloid beta (half life $\sim 2$ h), tau protein ($\sim 11$ days), and alpha-Synuclein ($\sim 6$ h) are all related to neurodegenerative disorders and are present in the interstitial space surrounding neurons^20–22^. Consistent with there being a spectrum of clearance time constants, experiments on chronic sleep restriction show continued worsening of performance and cognitive impairment on timescales of at least weeks^23–25^. All these factors point to the existence of some waste products with clearance times on the order of a week or longer, tau protein being a known example.

In some degenerative states, proteins such as tau and alpha-Synuclein are known to accumulate to abnormal levels. Notably, sleep abnormalities are widely observed in Alzheimer’s disease, often appearing decades before manifestation of cognitive symptoms^9,10^. Alzheimer’s disease is associated with well known cognitive impairments and other conditions also display cognitive decrements in association with reduced clearance rates—including reduced behavioral alertness^23–25^, natural brain aging, and traumatic brain injury^11,26,27^. Accumulation of waste chemicals can further slow clearance, which can lead to subarachnoid hemorrhage, acute ischemia, and multiple micro-infarctions^28,29^. It is thus likely that the buildup of abnormal deposits of waste products may itself interfere with clearance in a positive feedback loop in these conditions. Conceivably, some conditions may even push the obstruction caused by accumulation of waste beyond a threshold for runaway buildup.

In the present work we extend our prior model that includes single-timescale clearance of a generic waste product to allow for a spectrum of production and clearance rates for a variety of such products. We also incorporate the higher clearance rate in sleep and impairment of clearance by waste buildup. We first calibrate the resulting model by requiring that it correctly reproduce published experimental results on normal sleep-wake cycles, sleep deprivation and recovery, and performance decrements during chronic sleep restriction (CSR). The calibrated parameters are then used to verify the model’s predictions against other experimental protocols. Finally, regimes of runaway waste buildup and their potential relationship to Alzheimer’s disease are explored.

## Theory

In this section we first briefly summarize the basic arousal dynamics model that underlies the present work. This focuses on the homeostatic aspects and we refer the reader to^30–36^ for a fuller description of the circadian sector of the model^33^, which is not changed in the present work. Then we develop our new homeostatic clearance model and explore its predicted dynamics.

### Arousal dynamics model

**Figure 1:**
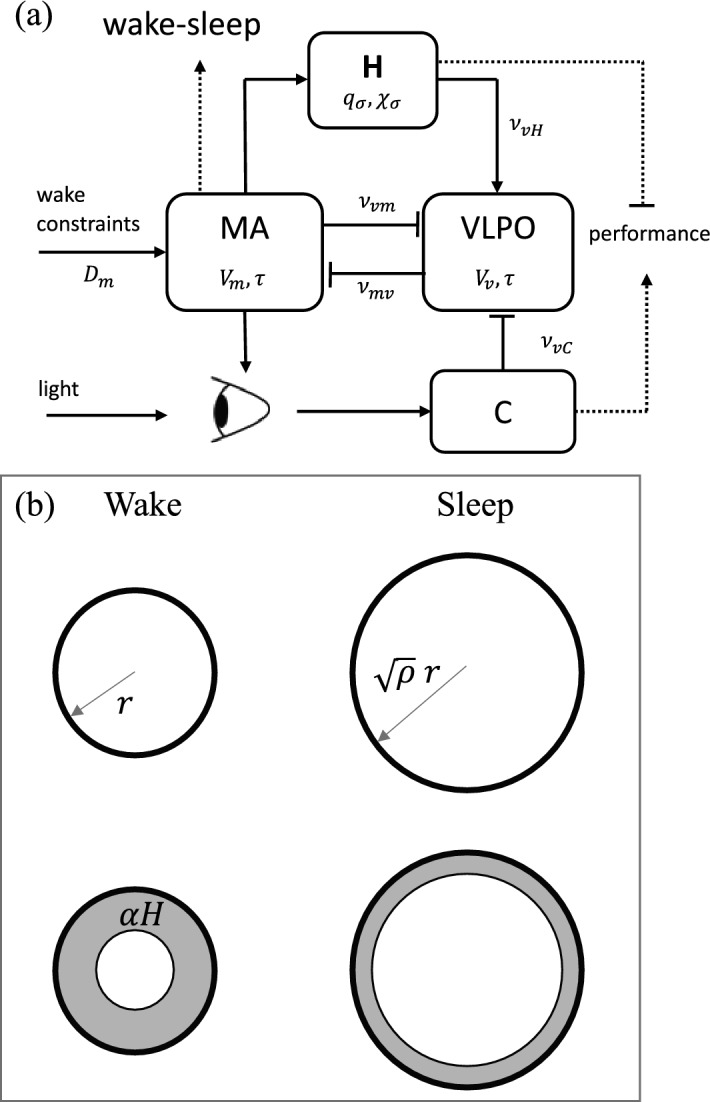
Model schematics. (**a**) Model of arousal dynamics incorporating key interactions between the homeostatic drive *H*, circadian drive *C*, and the sleep- and wake-active neuronal populations, VLPO and MA nuclei. Bar-headed lines indicate inhibitory connections, while the arrows show excitatory action. Dotted lines show relevant model outputs—sleep times and alertness. Constraints, such as sleep restriction are incorporated through action of the MA nuclei. See text for parameter descriptions. (**b**) Schematic cross section of an interstitial void during wake and during sleep with unobstructed flow (top) and obstructed flow (bottom) due to build up of waste products, shown shaded. The void radius increases during sleep by a factor of $\sqrt{\rho }$. In the absence of clearance, the area $\alpha H$ obstructed due to the effects of waste products is the same during wake and sleep.

Figure 1a summarizes the key features of the model. The dynamics of the sleep-wake switch arise from the mutual inhibition of monoaminergic (MA) nuclei that promote wakefulness and the ventrolateral preoptic (VLPO) nucleus, which promotes sleep; the inhibitory coupling ensures that only one population at a time can have high activity^37^. The mean soma potentials $V_m$ and $V_v$ of these two populations (*m* denotes MA and *v* denotes VLPO) are governed by the following equations:

1 $$\begin{aligned} \tau \frac{dV_v(t)}{dt}= & {} \nu _{vm} Q_{m}(V_m) - V_v(t) + D_v, \end{aligned}$$

2 $$\begin{aligned} \tau \frac{dV_m(t)}{dt}= & {} \nu _{mv} Q_{v}(V_v) - V_m(t) + D_m, \end{aligned}$$

where $\tau$ is a time constant, $Q_m$ and $Q_v$ are the mean neuronal firing rates in those populations, which are described by sigmoidal functions of $V_m$ and $V_v$. The couplings to *m* from *v* and to *v* from *m* are written as $\nu _{mv}$ and $\nu _{vm}$, respectively. We write the total external drive acting on the VLPO as $D_v$ and on the MA nuclei as $D_m$.

The activity of the *m* and *v* populations is regulated by the homeostatic (*H*) and circadian (*C*) drives, which are responsible for the timing of the sleep-wake transitions. The drives *C* and *H* act on the VLPO via the drive

3 $$\begin{aligned} D_v = \nu _{vC}C(t)+\nu _{vH}H(t)+A_v, \end{aligned}$$

where $\nu _{vC}$ and $\nu _{vH}$ are coupling coefficients with negative (inhibitory) and positive (excitatory) signs respectively, and $A_v$ is the drive to the VLPO from other neural populations. The circadian drive *C* arises from a dynamic circadian oscillator in the suprachiasmatic nucleus, which is entrained by the light-dark cycle^33,35^. This drive has been extensively documented in the references cited, so we do not discuss it here; values for all its parameters and temporal profile can be found in^33^. Sleep deprivation and sleep restriction are simulated by keeping the MA population in the wake state by adding a wake effort to the constant external input $D_m$, with detailed equations provided in^33^.

Our main concern in the present work is the homeostatic drive, which has been argued to reflect the accumulation of waste products during wake and their decline during sleep^3,6,7,10,38^. The buildup of waste products *H* has been modeled by

4 $$\begin{aligned} \frac{dH(t)}{dt} = q_{\sigma }-\chi _\sigma H(t). \end{aligned}$$

Here, *H*(*t*) is the effect of the species on sleep drive set to be dimensionless; $q_\sigma$ is the production rate in a state $\sigma$ where $\sigma =W$ denotes wake and $\sigma =S$ denotes sleep (the SI units of $q_\sigma$ are s$^{-1}$, although it is sometimes convenient to work in units of h$^{-1}$ and measure time in h); and $\chi$ is a clearance rate that was found to be $(0.5 {-} 1.7)\times 10^{-6}$ s$^{-1}$ (0.017–0.06 h$^{-1}$) in normal states^33^. The above model has successfully reproduced a range of experimental phenomena, including sleep times^30,31^, sleep propensity^33^, melatonin dynamics^35^, alertness^34^, response to the ambient light spectrum^36^, and real-life shiftwork^39^.

### Generalized homeostatic clearance

To develop the new model, we first generalize the homeostatic Eq. (4) to allow *H* to reflect the net effect of multiple chemical species, labeled *i*, with

5 $$\begin{aligned} H(t)=\sum _{i=1}^{N} H_i(t) \end{aligned}$$

We allow each waste species to have its own generation rate $q_{\sigma i}$ and clearance rate $\chi _{\sigma i}(t)$. These generalizations yield

6 $$\begin{aligned} \frac{dH_i(t)}{dt} = q_{\sigma i}-\chi _{\sigma i}(t) H_i(t). \end{aligned}$$

We now consider the effect of accumulation of waste products on fluid flow and waste clearance using a simple model in which uncleared waste obstructs the voids, either directly or by causing neighboring cells to swell. At small levels of accumulation the reduction in clearance rate will be approximately linear in the amount of accumulation, regardless of the clearance mechanism (although the relationship of the coefficient of the linear term to the underlying physics will be different). However, for tissue to survive, some clearance must continue, so $\chi _{\sigma i}$ must remain positive. Hence, we make the approximation

7 $$\begin{aligned} \chi _{\sigma i}(t)=\frac{\chi ^{(0)}_{\sigma i}}{1+\sum _j\beta _{\sigma j} H_j(t)}, \end{aligned}$$

where the blocking coefficient $\beta _{\sigma j}\ge 0$ parameterizes the contribution of $H_j(t)$ to the obstruction of clearance. This relationship captures the first-order modifications to $\chi$ but we expect it to be only qualitatively correct at large *H*.

Equation (7) has some relevant limiting cases. The first occurs when waste buildup is small and there is negligible impediment to flow. Then all $\beta _{\sigma j}$ are zero, $\chi _{\sigma i}(t)=\chi _{\sigma i}^{(0)}$, and the species evolve independently. This case corresponds to normal healthy sleep-wake cycles. The second special case is relevant at high waste buildup levels dominated by one product, which we designate by $j=1$ without loss of generality. In this case

8 $$\begin{aligned} \chi _{\sigma i}(t)=\frac{\chi ^{(0)}_{\sigma i}}{1+\beta _{\sigma 1} H_1(t)}, \end{aligned}$$

and the clearance rates of all species diminish as $H_1$ increases. The relevance of this case will become apparent below where we see that runaway of species concentration is possible, with likely dominance of one species.

If we consider the schematic cross sections of an interstitial void in Fig. 1b, we can estimate the dependence of $\chi _{\sigma i}$ and $\beta _{\sigma i}$ on void volume; this argument is unchanged if the voids are not circular, so as long as they are two-dimensional in cross-section (rather than being one-dimensional slits or fractals). First, we note that voids extend throughout the cortical gray matter, which does not change thickness significantly between wake and sleep, so the total void length and tortuosity cannot change significantly, consistent with experiment^8^. Hence, the observed change in each void’s volume must be accommodated chiefly via changes in its cross sectional area *A* due to neighboring cells expanding and contracting. Another effect that could be incorporated into this picture is that of changes in the outer radii of voids due to degenerative conditions that change brain volume—e.g., as is seen in Alzheimer’s disease.

For smooth flow through a tube of two-dimensional cross section the flow rate scales as $A^2$, as in the idealized case of Poiseuille flow in a cylindrical tube but with different proportionality^40^. So if the interstitial volume increases by a factor of $\rho$, the area increases by $\rho$ and the flow rate increases by $\rho ^2$, so

9 $$\begin{aligned} \chi _{Si}^{(0)}=\rho ^2\chi _{Wi}^{(0)}, \end{aligned}$$

where *S* denotes sleep and *W* wake. This scaling is consistent with the relationship between the observed volume increase of about 1.6 in sleep being correlated with a clearance rate increase of around 2.56^8^, as discussed in the Introduction. However, aside from this, the succeeding argument would not be changed significantly if mechanisms such as diffusion operated instead, except that $\rho ^2$ would be replaced by $\rho$, resulting in moderate quantitative changes in the dynamics described below, but not qualitative ones.

If obstruction of flow due to buildup of waste products is due to filling of an area $\alpha _i H_i$ with waste, the flow rate is reduced by a factor of roughly $(1-\sum _i\alpha _{Wi}H_i/A_W)^2$ in wake and $(1-\sum _i\alpha _{Si}H_i/A_S)^2$ in sleep. If we write these factors to first order as $(1+\sum _i\beta _{Wi}H_i)^{-1}$ and $(1+\sum _i\beta _{Si}H_i)^{-1}$, respectively, this is consistent with

10 $$\begin{aligned} \beta _{Si}=\beta _{Wi}/\rho . \end{aligned}$$

The increase in $\chi _{\sigma i}^{(0)}$ and the decrease in $\beta _{\sigma i}$ act in concert to enhance clearance during sleep. By solving the above equations find that the fraction of void blockage during wake is

11 $$\begin{aligned} \frac{\sum _i\alpha _{Wi} H_i}{A_W}= & {} 1-\frac{1}{\sqrt{1+\sum _i\beta _{Wi} H_i}}\approx \frac{1}{2}\sum _{i}\beta _{Wi}H_i, \end{aligned}$$

where the rightmost expression applies for small blocking fractions; analogous relationships hold in sleep.

### Dynamics of one species

Here we elucidate the dynamics predicted by the homeostatic clearance Eqs. (5) and (6) in a fixed state of arousal, including cases with nonzero $\beta _i$, we begin with the case of single species. The details of the mathematical derivations can be found in the Supplementary Materials.

In a fixed arousal state Eqs. (6) and (7) exhibit approach to a fixed point at

12 $$\begin{aligned} H_{\sigma i}^s= & {} \frac{q_{\sigma i}}{\chi ^{(0)}_{\sigma i}-q_{\sigma i}\beta _{\sigma i}}, \end{aligned}$$

provided $H^s_{\sigma i}>0$. However, if

13 $$\begin{aligned} q_{\sigma i}>\chi ^{(0)}_{\sigma i}/\beta _{\sigma i}, \end{aligned}$$

there is no fixed point and buildup of $H_{\sigma i}$ runs away, asymptoting to a linear increase as the second term in the denominator comes to dominate, canceling out the $H_i$ dependence on the right hand side of Eq. (6).

**Figure 2:**
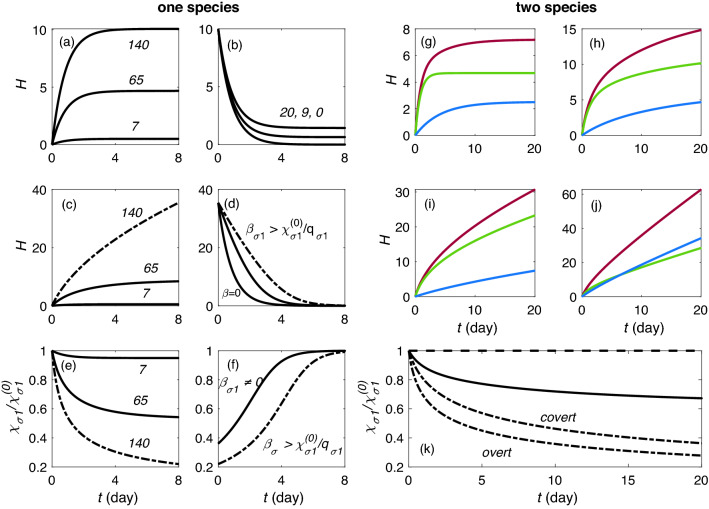
Homeostatic dynamics with one and two species in a fixed arousal state with and without runaway. One species dynamics are shown in (**a**)–(**f**). (**a**) Rising $H_1$ and $\beta _{\sigma 1}=0$ for production rates $q_{\sigma 1}=7\times 10^{-6}, 65\times 10^{-6},$ and $140\times 10^{-6}$ s$^{-1}$, as labeled in units of $10^{-6}$. (**b**) Falling $H_1$ and $\beta _{\sigma 1}=0$ for $q_{\sigma 1}=20\times 10^{-6}, 9\times 10^{-6},$ and 0 s$^{-1}$ from top to bottom. (**c**) Rising $H_1$ and $\beta _{\sigma 1}=0.1$ for the same values of $q_{\sigma 1}$ as in (**a**), with runaway case indicated with dot-dashed lines. (**d**) Falling $H_1$ for $\beta _{\sigma 1}=0.05, 0.1, 0$, from top to bottom. (**e**) Normalized clearance rate vs. *t* for the cases in (**c**), with runaway case indicated with dot-dashed lines. (**f**) Normalized clearance rate vs. *t* for the cases in (**d**); for $\beta _{\sigma 1}=0$ this ratio is 1 throughout so it is not shown. Homeostatic rise using two-species drive during wake is shown in (**g**)–(**j**). Fast clearance $H_1$ is shown in green, slow clearance $H_2$ in blue, and total *H* in red. (**g**) State with $\beta _1=\beta _2=0$ and $H_1$ and $H_2$ starting from zero. (**h**) Same as in (**g**) but $\beta _1=0.05$ and $\beta _2=0.15$. (**i**) Overt runaway state with $\beta _1=0.15$ and $\beta _2=0.15$. (**j**) Covert runaway. (**k**) Clearance dynamics for two species with $\beta _1=0.15$ and $\beta _2=0.15$ in dot dashed line, $\beta _1=0.05$ and $\beta _2=0.15$ in solid line, and $\beta _1=\beta _2=0$ in dashed line. In the covert runaway case $q_{\sigma 1}=65\times 10^{-6}$ s$^{-1}$ and $q_{\sigma 2}=30\times 10^{-6}$ s$^{-1}$; in all other cases $q_{\sigma 1}=65\times 10^{-6}$ s$^{-1}$ and $q_{\sigma 2}=7\times 10^{-6}$ s$^{-1}$.

To illustrate the dynamics of the model for a single species in a fixed arousal state (wake or sleep), we set generation and clearance rates to fixed values. Figure 2a–f show different cases for $\chi _{\sigma 1}^{(0)}=1.4\times 10^{-5}$ s$^{-1}$ with $q_{\sigma 1}=7 \times 10^{-6}, 65\times 10^{-6},$ and $140\times 10^{-6}$ s$^{-1}$. Solutions of Eq. (6) for a fixed arousal state with $\beta _{\sigma _1}=0$ are shown in Fig. 2a. In each case, starting from the initial value of $H_1(0) = 0$, $H_1$ rises steeply, then saturates exponentially on a timescale of $1/\chi _{\sigma 1}^{(0)}$ as it approaches the stable fixed point in Eq. (12); such dynamics is expected in wake. Figure 2b shows cases in which $H_1(0)$ is higher than the fixed point value; here we see that $H_1(t)$ decays exponentially toward the fixed point when $q_{\sigma 1}$ has low values of $20 \times 10^{-6}, 9\times 10^{-6},$ and 0 s$^{-1}$, with dynamics more representative of sleep.

In Fig. 2c we consider the same cases as in Fig. 2a, but with $\beta _{\sigma _1} = 0.1$. In each case, starting from the initial value of $H_1(0) = 0$, $H_1$ rises and either approaches the stable fixed point in Eq. (12) or continues to rise indefinitely, depending on whether $q_{\sigma 1}$ is smaller than the threshold $\chi ^{(0)}_{\sigma 1}/\beta _{\sigma 1}$ or not. With $q_{\sigma 1}=7\times 10^{-6}$ and $65\times 10^{-6}$ s$^{-1}$ the system approaches fixed points with $H_1=0.5$ and 8.4, respectively, in accord with Eq. (12), which yields higher values than when $\beta _{\sigma 1}=0$ and there is no obstruction (see also Supplementary Material S1). For $q_{\sigma 1}=140\times 10^{-6}$ s$^{-1}$, there is no longer a stable fixed point, the runaway condition (13) is fulfilled, and we see an asymptotically linear rise of $H_1$. Figure 2d shows cases in which $H_1(0)$ is higher than the fixed point value for three different $\beta _{\sigma 1}$. While $H_1(t)$ decays monotonically toward the fixed point at $q_{\sigma 1}=0$ in all cases, void obstruction slows clearance for $\beta _{\sigma 1}\ne 0$.

To explore how the accumulation of waste products obstructs fluid flow and consequently reduces the clearance rate, Fig. 2e illustrates the dynamics of clearance in the same cases as in Fig. 2c, in accordance with Eq. (7). When $\beta _{\sigma 1}=0$ $\chi _{\sigma 1}/\chi ^{(0)}_{\sigma 1} =1$ at all times. In the other cases, as $H_{1}(t)$ rises, this ratio decreases as $1/[1+\beta _{\sigma 1} H_{1}(t)]$. For the cases with valid fixed points, the clearance asymptotes to values of 0.94 and 0.54 for $q_{\sigma 1}= 7\times 10^{-6}$ and $65\times 10^{-6}$ s$^{-1}$, respectively. However, in the runaway case where $q_{\sigma 1}$= $140\times 10^{-6}$ s$^{-1}$ and $H_{1}(t)$ keeps increasing, the clearance ratio decreases monotonically toward zero and total void blockage. The dynamics of clearance for the cases in Fig. 2d is shown in Fig. 2f. Due to their different $\beta _{\sigma 1}$, their clearance values differ at $t=0$. Over time $H_1(t)$ decays and the clearance recovers to its unobstructed value; as before, this is fastest for small $\beta _{\sigma 1}$.

### Dynamics of two species

We now consider Eqs. (6) and (7) for two species ($i=1,2$), where we assume that species 1 has the larger effect on blockage, at least initially. Details of the derivation are found in Supplementary Materials S2. In essence, a fixed point is again found, unless

14 $$\begin{aligned} q_{\sigma 1}+q_{\sigma 2}>\frac{\chi ^{(0)}_{\sigma 1}+\gamma \chi ^{(0)}_{\sigma 2}}{\beta _{\sigma 1}+\gamma \beta _{\sigma 2}}, \end{aligned}$$

which generalizes Eq. (13). The fixed point level of $H_{\sigma 2}$ is increased by the presence of other species (in this case species 1), which tend to impede its clearance. If a fixed point does not exist, asymptotically linear runaway again occurs with the ratio of concentrations approaching a limit $\gamma$

15 $$\begin{aligned} \gamma =\lim _{t\rightarrow \infty }\frac{H_{\sigma 2}(t)}{H_{\sigma 1}(t)}, \end{aligned}$$

with the second species’ dynamics slaved to those of the first if the first dominates (see the Supplementary Materials for detailed expressions); we term this *overt runaway*.

To illustrate the dynamics of the model for two species in a fixed arousal state, we set $\chi _{\sigma 1} = 1.4\times 10^{-5}$ s$^{-1}$, $\chi _{\sigma 2} = 0.27\times 10^{-5}$ s$^{-1}$, $q_{\sigma 1}=65\times 10^{-6}$ s$^{-1}$, and $q_{\sigma 2}=7\times 10^{-6}$ s$^{-1}$. The resulting dynamics of *H* are shown in Fig. 2g–j. The case of rising *H* with $\beta _1=\beta _2=0$ is shown in Fig. 2g. Both $H_1(t)$ and $H_2(t)$ start from 0, rise toward stable fixed-point values of 4.7 and 2.5, respectively, in accord with the detailed expressions in Supplementary Materials S2. Consequently, their sum *H*(*t*) reaches 7.2. For values $\beta _{\sigma 1}=0.05$, $\beta _{\sigma 2}=0.15$, the dynamics are similar to those for $\beta _{\sigma 1}=\beta _{\sigma 2}=0$, but reaching higher fixed points for both species, as shown in Fig. 2h due to the slower clearance. It is clear that in both Fig. 2g,h *H*(*t*) reaches a fixed stable point after an initial rise, but the values with nonzero $\beta _{\sigma i}$ are higher, in accord with Supplementary Material S2. In Fig. 2i the system saturates at $H_1=12$ and $H_2 = 6.4$ because the runaway condition (14) is not satisfied. In these cases and for small $\beta _{\sigma i}$ the dynamics of the individual species are nearly independent. However, runaway occurs if $\beta _{\sigma 1}$ increases beyond 0.14. In this case it is not only species 1 that runs away approximately linearly in time but also species 2, which is slaved to it, as shown in Fig. 2j. As $\beta _{\sigma 1}$ becomes larger, the ratio $H_{\sigma 2}/H_{\sigma 1}$ approaches a constant value of $\gamma =0.47$, which is in accord with the expression in Supplementary Material S2. The clearance dynamics for Fig.  2i,j is shown in Fig. 2k. In the case with $\beta _{\sigma i}$ lower than runaway threshold for both species, the clearance deviates from $\chi ^{(0)}_{\sigma i}$; however, in the overt runaway case where $\beta _{\sigma 1}$ has a large value, the clearance falls toward zero.

If the runaway condition (14) is satisfied for species 2 but not for the initial dynamically dominant species 1, $H_2$ cannot saturate and we term the situation *covert runaway*. In this case, species 2’s concentration will increase approximately linearly in time without changing the system dynamics very much at first. However, once $H_2$ becomes comparable with $H_1$ it will affect sleep-wake cycles, and when $\beta _2H_2$ becomes of order unity, its contribution to void blocking will become dynamically significant and may eventually dominate; in any event, the continued rise in $H_2$ will push $H_1$ up in this case. We conclude that significant contributions from species 2 to disturbances to sleep-wake cycles may occur.

### Cycle-averaged dynamics

The results in the previous subsections are for a fixed arousal state $\sigma$. In most situations, there is a daily alternation between sleep and wake and we are interested in the long-term evolution of waste product levels over many such cycles when they are well above normal levels (in normal states it suffices to study a single periodic cycle). It is thus useful to average the key results over a complete sleep-wake cycle, assuming that a fraction $f_W$ of the cycle is in the wake state and the rest is in sleep. The mean level of *H* changes little between sleep and wake under severe chronic sleep restriction or runaway states. Averaging Eq. (6) over time and denoting means by overbars gives

16 $$\begin{aligned} \frac{d\overline{H_i}}{dt}= & {} \overline{q_i}-\overline{\chi _i}\overline{H}_i, \end{aligned}$$

17 $$\begin{aligned} \overline{x}= & {} f_Wx_W+(1-f_W)x_S, \end{aligned}$$

where Eq. (17) applies for any quantity $x_\sigma$. Hence, at large $\overline{H}_i$ with $\beta _iH_i\gg 1$

18 $$\begin{aligned} \frac{d\overline{H_i}}{dt}\approx \overline{q_i}-\frac{\chi _{Wi}^{(0)}}{\beta _{Wi}}\left[ f_W+\rho ^3(1-f_W)\right] ; \end{aligned}$$

cycle-averaged runaway occurs when the right side of Eq. (18) is positive.

The two terms in the square brackets in (18) are proportional to the contributions of clearance during wake and sleep, respectively. We see that clearance during sleep dominates if

19 $$\begin{aligned} f_W<\frac{\rho ^3}{1+\rho ^3}, \end{aligned}$$

which corresponds to waking times of less than about 19 h per day for $\rho =1.6$. More generally, we expect $f_W$ to decrease at high $\overline{H}$ in response to increased homeostatic sleep pressure. We note that it is possible for the runaway condition to be satisfied in wake, but not in sleep, since the ratio $q_{\sigma i}\beta _{\sigma i}/\chi _{\sigma i}^{(0)}$ is decreased by a factor of $\rho ^3$; in this case sleep is mandatory to achieve system stability against runaway. Since sleep is seen in all mammals, it is likely that this is the regime in which the brain actually operates and; indeed, the shortest-sleeping mammals such as elephants still spend around 3 h per day asleep^41^. Note that if diffusion, rather than advection, dominates clearance $\rho ^3$ is replaced by $\rho ^2$ in Eq. (19) and the boundary implied by this equation is around 17 h of wake per day.

## Results

In this section we calibrate the model parameters by adjusting them to fit the model’s predictions to published experimental data from a few specific experiments. Having reproduced those results, we verify the model predictions against other published experiments without further adjusting the parameters. Then we explore the consequences of runaway in cases of abnormal waste production or clearance.

### Model calibration

We calibrate the generalized model against normal sleep-wake cycles, sleep deprivation and recovery, and chronic sleep restriction (CSR). We do not expect perfect agreement given the simplicity of the model, but we aim for results that are semiquantitatively accurate and sufficient for wider applications in later sections.

Our generalized model needs to be able to explain the long-term build-up of deficits in chronic sleep restriction (CSR) while still accounting for normal sleep-wake dynamics, where predictions from earlier, more basic, models have been extensively verified. We also wish to incorporate an improved approximation to the production rates in sleep. This means that the parameters of the extended model need to be fitted to suitable calibration data from these conditions before being used to predict dynamics in new situations. We first attempt these tasks under the simplest assumption, as used in prior work, that only one species’ dynamics need be tracked—which may be an “effective species” whose properties are weighted averages of several species that jointly dominate the dynamics. We know this approach enables normal sleep dynamics and total sleep deprivation to be tracked^1,2^, and aim to determine whether CSR dynamics can also be accounted for via the poorer homeostatic clearance during increased wake. After exploring the single-species case, we repeat the process with the addition of a slow-clearing species to model the dynamics of tau and other long-lived waste products.

The four model extensions made for one species here are to: (i) incorporate the homeostatic function, with a time constant (inverse of clearance rate) expected to be in the range previously found by fitting the model to experimental data^33^, and also by accounting for chronic sleep restriction; (ii) incorporate nonzero $\beta _{\sigma i}$ to account for void obstruction; (iii) allow for the approximately 60% increase in total void volume in sleep via its effects on the $\chi _{\sigma i}^{(0)}$ and $\beta _{\sigma i}$ via Eqs. (9) and (10) for $\rho =1.6$, so only wake values need be specified separately; and (iv) set the homeostatic production rate in sleep to 0.8 times its rate in wake, in accord with experiment^42^, instead of to zero as in^30,31,43^. This last point is justified because we expect the higher clearance rate in sleep in the extended model to offset the higher sleep production rate, leading to similar outcomes from a more physically realistic basis. Specifically, we write the production rate $q_{\sigma i}$ as the product of a dimensionless constant $\eta _i$ and the mean cortical neural firing rate $Q_\sigma$ (SI units s$^{-1}$), with

20 $$\begin{aligned} q_{\sigma i}= \eta _{i} Q_{\sigma }, \end{aligned}$$

with $Q_W=7$ s$^{-1}$^43^. Other parameters of the model, including its circadian aspects are left unchanged from those in prior work^33–36^.

We aim to reproduce three sets of experimental conditions in order to calibrate the model before applying it to predict other dynamics in later sections. These are as follows:

1. Without applying any constraints on sleep opportunity, the model must reproduce normal sleep-wake dynamics with approximately 8.2 h as the optimal sleep duration^23^ with a typical range of 7.9–8.5 h and a sleep onset time between about 2230 and 0030.
2. The model must reproduce the right subsequent sleep duration after a long period of total sleep deprivation (SD)^44^. Specifically, the first sleep bout that follows a long period of total sleep deprivation is usually 12–15 h even after a several-day deprivations^44^.
3. The model must reproduce the CSR dynamics from Van Dongen et al.’s^23^ study with 3 baseline days (8 h sleep opportunity) followed by either 3 nights of total sleep deprivation or 14 nights of sleep restriction (0, 4, 6, or 8 h sleep opportunity per day, resulting in actual mean sleep durations of 0, 3.7, 5.5, and 6.7 h per day, respectively). This experiment compared waking neurobehavioral functions during sleep restriction with those for total sleep deprivation^23^. It used the psychomotor vigilance task (PVT) in which reaction times to stimuli in each 10 min test bout every 2 h were counted as attentional lapses when they exceeded 500 ms. The temporal regulation of sleep is governed by the homeostatic and circadian processes and this temporal regulation of sleep affects human neurobehavioral functions like sustained attention^34,45,46^. It has previously been shown^34^ that a fair approximation to the number of excess PVT lapses *p* relative to baseline at a given circadian phase is
  21 $$\begin{aligned} p=\lambda \Delta H, \end{aligned}$$

where $\Delta H$ is the deviation from baseline and the constant $\lambda$ is chosen by minimizing the root-mean-square difference between theory and experiment over the four conditions.

To assist in finding optimal parameters from dynamics, we define a qualitative goodness-of-fit parameter $\zeta$, which we minimize over the parameter space, as described in the Methods.

### One-species fit

**Figure 3:**
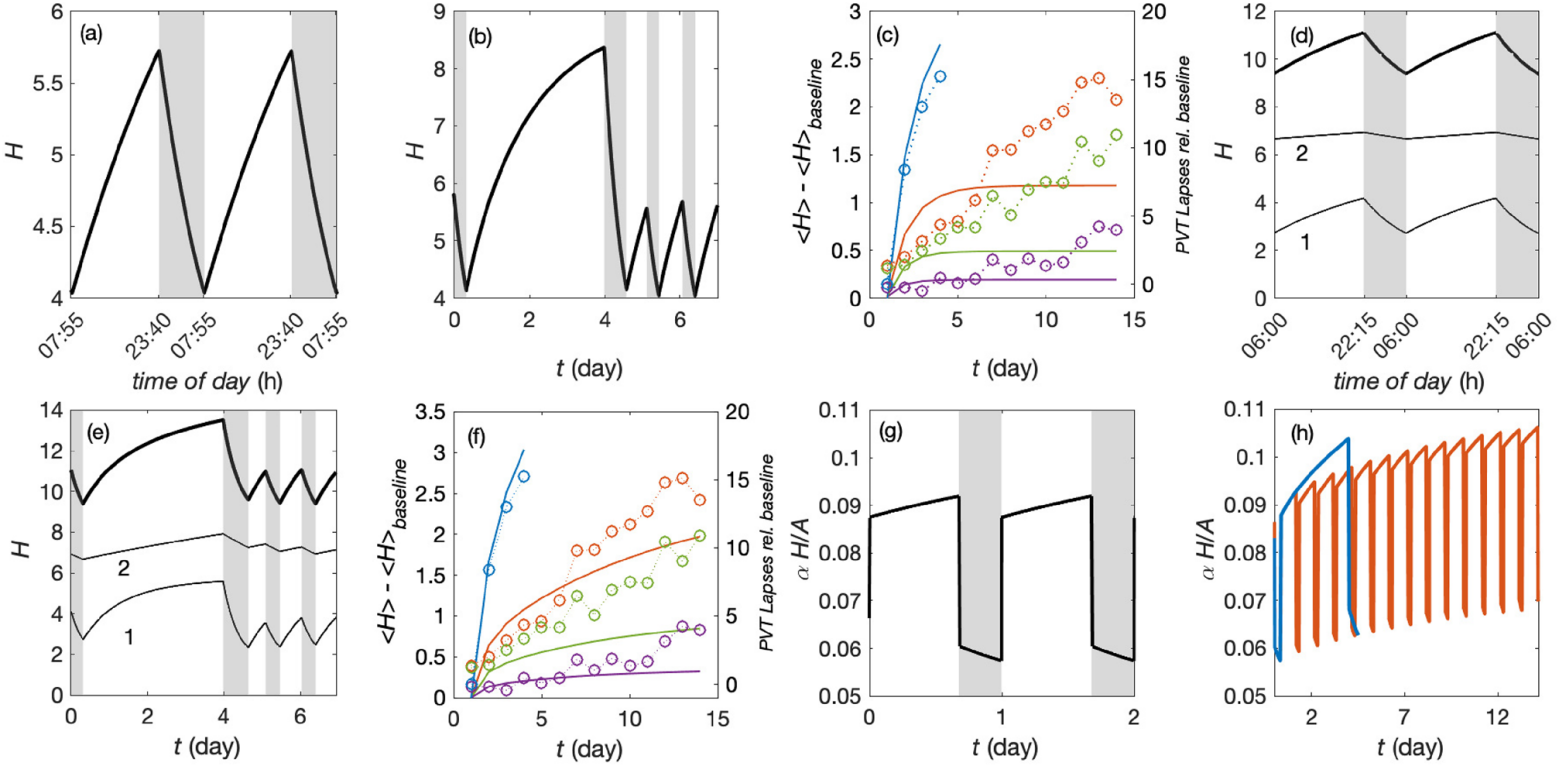
One and two species dynamics of *H* and void blocking fraction for the best-fit parameters in Table 1 for the calibration experiments. One species dynamics for (**a**) normal sleep; (**b**) sleep deprivation; and (**c**) chronic sleep restriction, cycle-averaged and normalized to the baseline days with blue showing sleep deprivation, orange 4 h sleep opportunity, green 6 h, and purple 8 h. Two species dynamics for (**d**) normal sleep, (**e**) sleep deprivation, (**f**) chronic sleep restriction, cycle-averaged and normalized to the baseline days, plotted as in (**c**). Open symbols show PVT lapse data from Van Dongen et al.’s^23^ study. Void blockage fraction for two species: (**g**) during normal sleep-wake cycles, and (**h**) during chronic sleep restriction from^23^. Blue, red, green, and purple indicate sleep deprivation, 4, 6, and 8 h sleep opportunity per day, respectively.

We obtain a simultaneous fit to normal sleep, SD, and CSR data by varying the parameters $\chi$, $\beta$, $\eta$, and $A_v$ to minimize $\zeta$ across the data sets discussed in the previous subsection. To do this, we employ the genetic algorithm function in the Matlab optimization toolbox^47^. This algorithm initially generates a population of 50–200 random points depending on the number of variables. The point with the smallest $\zeta$ is then selected to generate a fresh set of random points in its vicinity and the new point with the smallest $\zeta$ is then selected. These latter steps are repeated until $\zeta$ ceases to decrease significantly and the corresponding parameters are used as our estimate of the optimal values. The optimal one-species parameters found are shown in Table 1 and Fig. 3a–c show an example of *H* dynamics for the optimal parameters. We find 8.22 h sleep duration which starts at 2340 for normal sleep wake cycles without applying any constraints on sleep opportunity, as shown in Fig. 3a. After 88 h of sleep deprivation, 14.5 h of subsequent first night sleep is found, which is shown in Fig. 3b. Figure 3c shows the dynamics of *H* compared to the PVT lapses data for chronic sleep restriction. One sees that the model *H* saturates within about 5 days, whereas the experimental curves continue to rise, implying that the first two fits are close, but that CSR is poorly fitted and the long-term dynamics are not as well modeled as more rapid changes, apparently because the value of $\beta$ required to account for short-term dynamics is too small to impede clearance sufficiently at long times. This points to the need to include a second species with slower dynamics and larger blocking coefficient $\beta$.

### Two-species fit

The optimal two-species parameters found are shown in Table 1 and the dynamics are shown in Fig. 3d–f. The shorter clearance time is reduced by about a third relative to the one-species case to about 19 h, while the longer one is 9 days, within 20% of the value estimated for tau protein^22,48^. The blocking coefficient for species 2 is of the same order as for the one-species case, while for species 1 it is lower than in the one-species case, whose value needed to be higher to yield sufficiently slow clearance under CSR conditions. The production rate of the fast-clearing species is little changed from the one-species value, and that of the slow-clearing species is about 6 times lower. Finally, $A_v$ is shifted downward by 6 mV to compensate for the increased effect of mean homeostatic drive on the total external drive acting on the VLPO.

For the optimal two-species parameters, we find 7.7 h sleep duration which starts at 2215 for normal sleep wake cycles without applying any constraints on sleep opportunity, as shown in Fig. 3d, and consistent with the range of normal sleep duration. After 88 h of sleep deprivation, 15.8 h of subsequent first night recovery sleep is found, which is shown in Fig. 3e, also consistent with experiment^44^. The fast-clearing species saturates within the sleep deprivation period, whereas the slow-clearing species continues to build up. Figure 3e shows that the model *H* now has a behavior that is semiquantitatively in agreement with the experimental results, displaying in particular continued buildup over the full 14 days instead of quickly saturating. After 14 nights of sleep restriction, the *H* level for the 4 h condition reaches the same level as two nights of total sleep deprivation and the 6 h condition reaches approximately the same level as one night of total sleep deprivation. For the 8 h sleep condition *H* still increases across the simulation because the mean sleep duration is only 6.7 h as in the experiments, but this is slower than in the other cases.

It is noteworthy that PVT is known to be influenced by factors other than *H*^49^ so we do not expect exact agreement with PVT data. To explore the role of waste accumulation under normal and CSR conditions, the fractional blocking of voids is calculated from Eq. (11). Figure 3g shows the void blockage fraction in the two-species case under normal conditions. During wake the blockage reaches $9.2\%$ of the total void cross section, whereas during sleep after the full clearance it falls to $5.7\%$. The void blockage fractions for sleep deprivation and CSR are shown in Fig. 3h. At the end of sleep deprivation, void blockage reaches nearly $10.4\%$ of the total void cross section, which is close to the level of $10.6\%$ attained at the end of 14 days of 4 h sleep restriction, consistent with the similar PVT scores in the two cases. It is notable that the typical scale of normal daily variations in void blockage is less than $\pm 4\%$ of total void cross section from peak to trough and even multiday sleep deprivation or CSR produce departures of less than 4% of the total void cross section; this sets a scale against which other levels of blockage can be assessed.

**Table 1:** Model parameters.

Parameter	Symbol	Value	Units
Void ratio	$\rho$	1.6	–
Firing rate	$Q_{W}$	7	s$^{-1}$
**One-species fit**			
Sleep drive	$A_v$	$-3.0$	mV
Clearance time	$1/\chi ^{(0)}_{W}$	$1.06\times 10^5$	s
Blocking coeff.	$\beta _{W}$	0.026	–
Production coeff.	$\eta$	10.1$\times 10^{-6}$	—
**Two-species fit**			
Sleep drive	$A_v$	$-8.0$	mV
Clearance time	$1/\chi ^{(0)}_{W1}$	$0.71\times 10^{5}$	s
Blocking coeff.	$\beta _{W1}$	$2.8\times 10^{-3}$	–
Production coeff.	$\eta _1$	$9.4\times 10^{-6}$	–
Clearance time	$1/\chi ^{(0)}_{W2}$	$8.0\times 10^{5}$	s
Blocking coeff.	$\beta _{W2}$	0.029	–
Production coeff.	$\eta _2$	$1.65\times 10^{-6}$	–

Each line shows a parameter, its symbol, fitted value, and units in successive columns. Assumed homeostatic parameters are listed in the first block of the table. The second block shows parameters obtained from the one-species fit. The third block shows the corresponding two-species parameters. The full list of underlying circadian model parameters is found in^34^.

### Recovery from CSR

Having calibrated our two-species model against data, we now apply it to different experiments that embody aspects of sleep restriction and recovery without adjusting the parameters from those previously determined. This serves to further verify the model’s semiquantitative validity.

Belenky et al. studied CSR and post-CSR recovery in 66 subjects^24^. The first 3 days of their protocol required 8 h required time in bed (TIB) per day. From the fourth to tenth days, subjects underwent one of four conditions: 9 h required TIB (2200–0700), 7 h required TIB (2400–0700), 5 h required TIB (0200–0700), or 3 h required TIB (0400–0700). These comprised a sleep augmentation condition and three sleep restriction conditions. On the 11th to 13th days, subjects were again required to be in bed for 8 h, giving a recovery period. Average total sleep time (TST) over the 7 days of sleep restriction or augmentation were 7.93 h for the 9-h TIB group, 6.28 h for the 7-h TIB group, 4.66 h for the 5-h TIB group, and 2.87 h for the 3-h TIB group.

**Figure 4:**
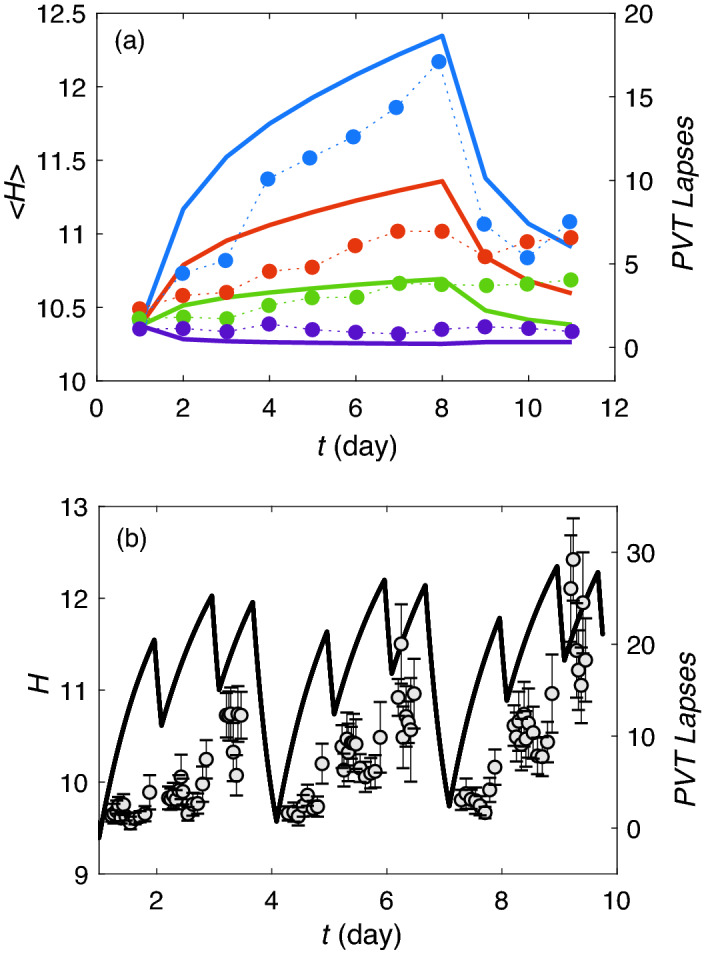
Model predictions for CSR protocols that were not used in parameter calibration. (**a**) Belenky et al.^24^ chronic sleep restriction and recovery, averaged across each cycle with two-species parameters from Table 1. Blue indicates 3 h of chronic sleep restriction, red 5 h, green 7 h, and purple 9 h. Filled circles show the experimental data. (**b**) St. Hilaire et al.^25^ chronic variable sleep restriction for two-species parameters from Table 1. Model prediction of *H* dynamics are shown with solid line. Experimental data are shown with gray circles.

The results shown in Fig. 4a indicate an increase in *H* for 3, 5, and 7 h TIB conditions and a decrease for the 9 h TIB condition, all of which accord with the PVT data. Sleep recovery is somewhat slower after the 3 h TIB condition than seen in the data, but faster after the 5 h TIB condition. The data show no recovery after the 7 h TIB condition, whereas our *H* level recovers towards the baseline level. The sleep duration after 9 h of sleep restriction also recovers to near the baseline after 3 nights. The TST in sleep recovery periods shows a similar trend to the data. Although an 8 h sleep recovery opportunity was given for three nights, the 3 h TIB condition data show approximately 7.5, 7, and 7 h TST for these nights; our corresponding result likewise shows the longest TST occurs on the first night of recovery with 8 h followed by 7.9 h for the next two nights for slightly more recovery sleep on average. The data after the 5 h and 7 h TIB conditions both show approximately 7, 7, and 6.5 h TST during recovery; our results again show the same downward trend over the three nights, but more sleep on average: 7.9, 7.9, and 7.8 h after the 5 h TIB condition and 7.8, 7.8, and 7.7 h after the 7 h TIB condition. The 9 h TIB condition data show nearly constant 7 h TST across all three nights, in agreement with the trend in our results, which again predict somewhat more sleep on average at 7.6 h. Thus the trends are reproduced by our model to within the uncertainties of the data, apart from a slightly higher average TST in all cases, which is likely the result of the fact that we have not refitted our parameters to these data, but use the values from the previous section.

### Intermittent CSR

Our second application of the calibrated model is to the work of St. Hilaire et al., who studied intermittent CSR, with periodic recovery opportunities, as an analog of common sleep schedules in which CSR occurs during the week, with recovery on weekends^25^. The protocol started with a baseline day of 10 h of scheduled sleep and 14 h scheduled wake. The first cycle of sleep restriction started on day 2, consisting of two 3-h TIB periods on successive days followed by one 10-h TIB period on day 4. This pattern was repeated twice more in blocks of 3 days (3 cycles). Our results in Fig. 4b indicate an increase in *H* after each cycle of sleep restriction despite the 10 h sleep opportunity, consistent with the long time constant of the slow-clearing species. However, the rate of degradation in PVT performance data in Fig. 4b following a 3-h sleep opportunity in the second and third cycles is steeper than the first cycle, whereas we predict roughly equal rates. This may indicate the presence of a third very-fast clearing species that enables greater recovery during sleep opportunities, and contributes to faster catch-up during subsequent periods of restriction; however, we do not pursue this possibility in the present work.

### Runaway buildup of tau protein

We now show that abnormalities in homeostatic processes, particularly rates of production and clearance of waste, can lead to drastically increased waste levels and void blockage, and even runaway buildup under some circumstances. We consider cases of both a sudden parameter change, as might happen after an injury, and of gradual evolution away from normal values, as may reflect progressive deterioration due to age, sleep disturbances, or other factors. In both cases we enforce a minimum of 10 h of wake per day ($f_W\ge 0.42$) to correspond to the wake-promoting effects of social and other pressures. Resulting buildup of tau protein occurs on timescales of many months to years, comparable to the onset period of Alzheimer’s disease, for example.

A key prediction of the preceding sections is that there is a threshold beyond which waste buildup runs away to levels far above normal. According to the cycled-averaged Eqs. (17) and (18), various effects could contribute to such an outcome: (i) an increase in the average rate of waste production $\overline{q_i}$ through an increase in its proportionality $\eta _i$ to firing rate in Eq. (20); (ii) an increase in the effectiveness of blocking by waste, reflected in the blocking coefficient $\beta _i$; (iii) a decrease in the value of $\rho$, corresponding to reduced dilation of interstitial voids during sleep; (iv) an increase of the fraction of time spent awake $f_W$; and/or (v) a decrease in the clearance rate $\chi _{Wi}^{(0)}=1/\tau ^{(0)}_W$. Continued runaway corresponds to the right side of the cycle-averaged Eq. (18) being positive. This implies threshold values for $\eta _2$, $\beta _2$, and $\tau _2$ approximately 10.5 times higher than their value in Table 1 if all the other parameters are held constant, whereas the thresholds for $\rho$ and $f_W$ cannot be reached for $\rho \ge 1$ and $f_W\le 1$.

**Figure 5:**
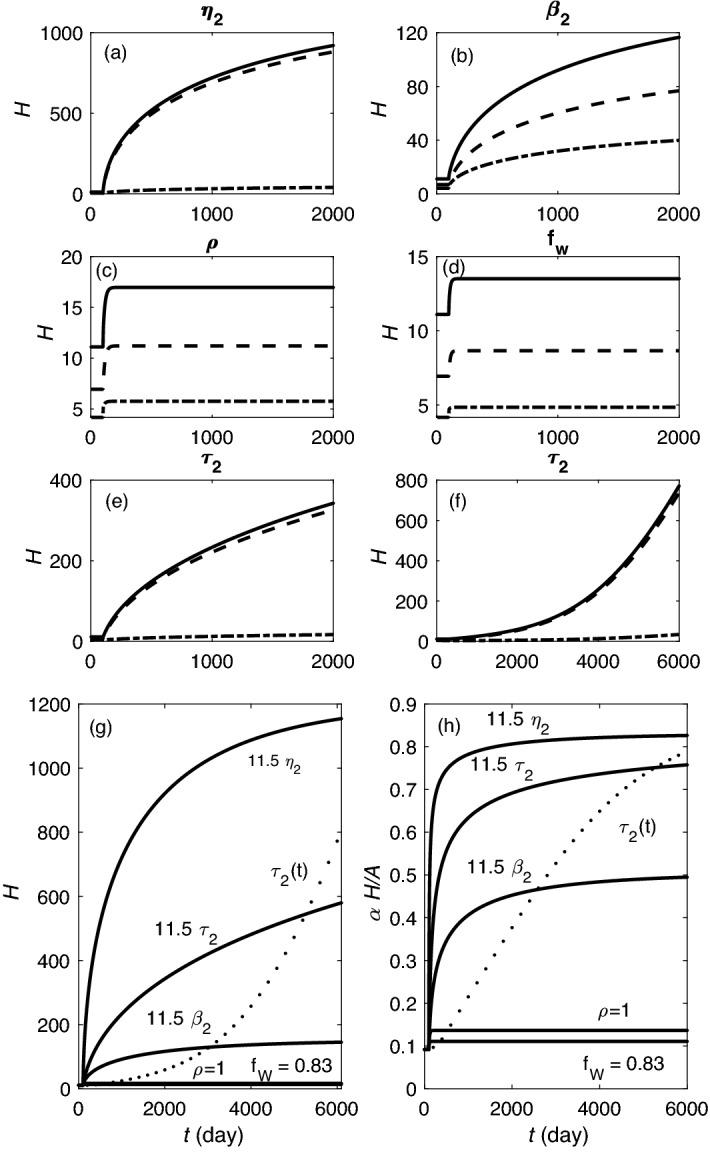
Runaway predictions for the two species. (**a**)–(**e**) Demonstrate the case of a sudden change of one parameter at $t=100$ days, as labeled: (**a**) $\eta _2$ is 11.5 times higher than the best-fit value from Table 1, (**b**) $\beta _2$ is 11.5 times the best-fit value, (**c**) $\rho = 1$, (**d**) $f_W$ is increased to 0.83 which indicates 20 h of wakefulness, and (**e**) $\tau _2$ is 11.5 times larger. In each case the dot-dashed line indicates $H_1$, the dashed line $H_2$, and the solid line, the total *H*. (**f**) Shows results for a gradual increase of $\tau _2$ over time, with an increase by a factor of 11.5 being reached at $t=3500$ days. (**g**) Comparison of total *H* for the conditions in (**a**)–(**e**), as labeled; dotted line shows case (**f**). (**h**) Corresponding void blockage fraction for the conditions in (**a**)–(**f**). All other parameters are set at their best-fit values from 1.

Figure 5 illustrates the effects of each of the above-mentioned changes in the parameters of the slow-clearing species. Runaway happens over the course of $\sim 5$ years when $\eta _2$, $\beta _2$, or $\tau _2$ is increased by a factor of 11.5 as shown in Fig. 5a,c,e, respectively, consistent with threshold values predicted from the cycle averages of quantities in Eq. (18). We find that, once runaway occurs after an increase in $\eta _2$, $\tau _2$, or $\beta _2$, the value of *H* increases to levels far above normal over a year or more. In contrast to these cases of runaway, Fig. 5c shows that setting $\rho =1$ to remove the expansion of interstitial voids during sleep leads to a rise in *H* followed by saturation. Likewise, Fig. 5d shows the same behavior if sleep is permanently restricted to only 4 h per day; this saturation is consistent with many people continuing to function reasonably normally under conditions of permanent CSR. In neither case is the predicted runaway threshold exceeded but the saturation values are well above those experienced in the cases of sleep deprivation or CSR discussed in earlier sections. It is important to mention here that the simple linear approximations made to the dynamics of *H* and its effects on the sleep-wake cycle will need correction at the very high levels of *H* attained in the cases of runaway, so the trends seen should be only treated as qualitative once *H* exceeds a few times the level found in CSR where the results have been verified against experiment.

In Fig. 5h we see that interstitial void blockage fraction rises rapidly in all cases considered, slowly approaching 100% asymptotically in runaway cases, and saturating at 12–14% in the cases when *H* saturates, well above the level seen after 14 days of CSR in Fig. 3h. In the runaway cases the blockage fraction after 5 years is 45% to 80%. The steepest increase in the blockage fraction occurs before 2000 days ($\sim 5$ years), after which it slows and reaches 50% to 82% at 6000 days ($\sim 16$ years), in accord with the square-root dependence seen in Eq. (11). Once our dynamically dominant second species runs away, the ratio $H_1/H_2$ approaches a limit $\gamma$ that is consistent with the expression in Supplementary Material S2 with the roles of species 1 and 2 reversed. This ratio converges to 0.04 when $\eta _2$ increases, and to 0.05 when $\tau _2$ increases in the simulations (Fig. 5a,e), and to 0.52 when $\beta _2$ increases (Fig. 5b). In each case, these values are bracketed by the predicted wake and sleep values, while the approximate cycle-averaged equation (15) predicts ratios of 0.06, 0.07, and 0.7, respectively. This behavior is evident in Fig. 5b where $H_1$ and $H_2$ levels increase together. Figure 5g shows all the cases in Fig. 5a–e superposed for comparison.

In many cases, the conditions that lead to runaway are gradual, rather than sudden, owing to ongoing deterioration due to aging or pathology. Hence, we consider an example in which $\tau _2$ increases linearly, reaching 11 times its original value after 10 years, then continuing to rise at the same rate. Figure 5f shows the resulting increase in *H*, which is slower than the sudden-onset case at first, but steadily accelerating, with the runaway threshold being reached at around 3500 days. Prior to runaway, the levels of $H_1$ and $H_2$ roughly follow the fixed point values given in Supplementary Materials S2 with the labels 1 and 2 reversed; however, because of time lags in the dynamics, the fixed point is never quite reached and $H_1/H_2$ stays at about 0.4 of the fixed-point value. During the first 300 days, $f_W$ falls to its imposed floor of 0.42 (10 h per day of waking) in an attempt to increase cycle-averaged clearance, in agreement with observed sleep increase in the leadup to Alzheimer’s dementia^50^. Beyond about 2500 days, as the runaway threshold is approached, the fixed point value of $H_2$ rises rapidly, as expected from Supplementary Materials S2, and $H_1/H_2$ decreases. Once $H_2$ runs away, it rises approximately linearly with time with $H_1$ slaved to it, as seen in Fig. 5f, with $\beta _{\sigma 2}H_{\sigma 2}\approx 10 \beta _{\sigma 1}H_{\sigma 1}$. The ratio $H_1/H_2$ approaches a limit of 0.04, whereas this value was found to be 0.07 using the approximate expression in Supplementary Materials S2. The fraction of void blockage reaches almost 80% Fig. 5h by the end of 16th year—again, far above the levels that correspond to significant performance decrements under sleep-deprivation or CSR conditions. Corresponding curves are also shown for the earlier cases in this frame.

The slope of *H*, once runaway occurs is 0.6 $\times 10^{-5}$ s$^{-1}$ in the simulation; given time lags, this approximate value is consistent with the cycle-averaged value of $\eta _{2} Q$ (0.9 $\times 10^{-5}$ s$^{-1}$) as $\chi _2$ increases, the voids become heavily blocked, and *dH*/*dt* approaches linear behavior.

In the cases above, occurrence of runaway required roughly an order of magnitude increase in the relevant parameter. However, we have also verified that if more than one parameter deviates from nominal, the necessary change can be much smaller. For example, if both the production rate and the clearance time constant increase by about a factor of 3.5, the combination suffices to cause runaway.

## Discussion

We have developed a biophysical model of homeostatic waste clearance in the brain and incorporated it in a model of arousal dynamics that also includes circadian effects. The key new features of the model are (i) higher glymphatic clearance of waste products from the brain in sleep than wake^7,8,10^, (ii) the effects of waste-product buildup on clearance, (iii) multiple time constants of waste clearance, and (iv) improved approximation of waste production rates in sleep.

This new model with two species allows us to explain the sleep-wake and cognitive dynamics observed at different time scales, from days in normal sleep-wake cycles and acute sleep deprivation, and weeks to years in chronic sleep restriction. Having determined parameters by fitting predictions to calibration data from normal arousal dynamics and CSR experiments, the model reproduces experimental results on recovery from sleep deprivation and sleep restriction and on intermittent sleep restriction, without further adjustment. This is the first model to combine these different temporal scales of sleep disturbances using physiological mechanisms at the level of the homeostatic drive. Several other models have described the dynamics of cognitive outputs, like PVT, in acute sleep deprivation and chronic sleep restriction based on prior sleep history, but they did not incorporate the underlying dynamic homeostatic drive or brain clearance mechanisms^51–55^.

Notably, when two species’ parameters are fitted to data, one has time constant of $\sim 20$ h and properties close to those previously found to account for normal sleep-wake cycles and total sleep deprivation, whereas the other has a characteristic clearance time of $\sim 9$ days, which is close to that for tau protein^22,48^. This strengthens the possible link to tau buildup in neurodegenerative disorders such as Alzheimer’s disease.

Under normal sleep-wake conditions, inferred waste levels and corresponding homeostatic drive oscillate around a mean value over the circadian cycle with around 7–8% obstruction of interstitial void volume on average (15% obstruction of flow), varying between about 9% at sleep onset to around 6% just before waking. Corresponding figures after several days of sleep deprivation or two weeks of CSR are around 14% volume obstruction. However, we predict the existence of a threshold for runaway waste buildup, beyond which the waste itself obstructs clearance so greatly that it leads to runaway of the homeostatic drive and eventual near-total obstruction of interstitial voids. These predictions are in line with the evidence that in neurodegenerative disorders sleep disturbances can often precede cognitive symptoms by several years or even decades^9^. Significantly, prior to this critical point the model predicts a rapid increase in steady-state levels of waste products, which could potentially signal proximity of the runaway and could be detected experimentally via assays or imaging.

We explored runaway tau buildup driven by either sudden injury or gradual onset of pathology, especially in the form of enhanced waste production and/or impaired clearance. Timescales proved to be on the order of months to years, comparable to those observed for the onset of Alzheimer’s symptoms, including cognitive decline which has been associated with the buildup of tau plaques and neurofibrillary tangles^56^. Our identification of the key parameters for waste buildup and runaway highlights potential targets for monitoring and therapeutic intervention.

In the present study we have considered temporal changes in brain clearance and have assumed the same clearance and obstruction mechanisms across the brain. However, neurodegenerative diseases like Alzheimer’s are often characterized by specific spatial pattern of where those chemicals aggregate and provoke brain abnormalities^57^. For example, Amyloid-$\beta$ pathology starts in the cortex and only propagates to subthalamic regions in late stages of the disease^58^, whereas tau pathology first occurs in the locus coeruleus and enthorinal cortex, whence it expands to limbic and isocortical regions^59^. Quantitative study of such spatial dynamics would require the present model to be generalized to explicitly incorporate these structures and their differing activity levels and clearance parameters.

Normal ageing is also associated with reduction of glymphatic clearance (by $\sim 80{-}90$% in aged mice)^7,10,11^ which slows down fluid flow in the brain and facilitates accumulation of debris that may further obstruct clearance and change sleep patterns. The model predicts that such changes would be observed, at least partly due to accumulated effect of sleep disturbances over many years, even below the runaway threshold. This degradation of clearance in aging may contribute to cognitive decline seen even in healthy old adults. Our model provides a tool for exploration of how sleep-wake dynamics earlier in life affect cognitive outcomes in old age and for design of interventions to slow down or prevent cognitive decline.

Overall, the model has demonstrated a wide range of successful predictions, and makes others for further experimental testing. We stress that more accurate modeling of very large homeostatic drives and their links to cognition will be necessary to further refine predictions. Other underlying issues that would bear further investigation include more detailed modeling of diffusion and advection through insterstitial voids, the inclusion of spatial variations in parameters, modeling different forms of tau in both extracellular and intracellular locations, and including in situ degradation of waste alongside clearance. Other directions for future work include: (i) Brain clearance process in sleep shows an endogenous circadian rhythm^60^, which has not yet been incorporated. This is relevant when considering restricted sleep at different times of day, such as that in shiftworkers and during jetlag. This should be considered in the future because shiftworkers have been reported to have higher incidence of dementia^61^, which may plausibly be partly due to reduction of brain clearance when sleep occurs at inappropriate circadian times. (ii) The possibility that short-lived chemical species may be responsible for rapid partial restoration of cognitive performance by naps should be explored. (iii) The bidirectional connections between sleep patterns and clearance should be considered in detail. In this work we studied how sleep patterns affect clearance dynamics and showed that obstruction tends to increase sleep duration, but the effects of changes in clearance on sleep-wake cycles during healthy ageing should also be explored^7,10^.

## Methods

### Goodness of fit parameter

To assist in finding optimal parameters from dynamics, we define a qualitative goodness-of-fit parameter $\zeta$, which we minimize over the parameter space, with

22 $$\begin{aligned} \zeta= & {} \zeta _N+\zeta _{\mathrm{SSD}}+\zeta _{\mathrm{CSR}}, \end{aligned}$$

where $\zeta _N$, $\zeta _{\mathrm{SSD}}$, and $\zeta _{\mathrm{CSR}}$ are contributions from fits to normal, subsequent sleep duration after sleep deprivation, and CSR experiments.

The contribution $\zeta _N$ in Eq. (22) is written as

23 $$\begin{aligned} \zeta _N=\frac{(d-d_0)^2}{(\Delta d)^2}+ \frac{(s-s_0)^2}{(\Delta s)^2}. \end{aligned}$$

This expression contains terms that penalize deviations of sleep duration *d* from the nominal $d_0=8.2$ h and sleep onset time *s* from the nominal $s_0=23.5$ h, with tolerances $\Delta d=0.15$ h and $\Delta s=1$ h, respectively, based on optimal sleep duration range 8.05–8.35 h and night sleep onset range 2230–0030 h.

We define $\zeta _{\mathrm{SSD}}$ in Eq. (22) to be

24 $$\begin{aligned} \zeta _{\mathrm{SSD}}=\frac{(R-R_0)^2}{(\Delta R)^2} \end{aligned}$$

where the score measures the deviation of recovery sleep duration *R* from the nominal $R_0=13.5$ h following 88 h of total sleep deprivation with tolerance $\Delta R=1.5$ h based on optimal sleep duration range 12–15 h.

The contribution $\zeta _{\mathrm{CSR}}$ is calculated from the rms deviations *m* between the estimated PVT measure *p* and the experimental PVT data, summed across the four experimental conditions of total sleep deprivation, and 4 h, 6 h, and 8 h of sleep opportunity per day, respectively. Hence, we define $\zeta _{\mathrm{CSR}}$ to be

25 $$\begin{aligned} \zeta _{\mathrm{CSR}}=\frac{1}{4(\Delta m)^2}\left( m^2_0+m^2_{4}+ m^2_{6}+m^2_{8}\right) , \end{aligned}$$

where the subscripts indicate the number of hours of sleep opportunity per day and we set $\Delta m=0.15$ to give similar weight to the normal and CSR contributions in Eq. (22).

## Supplementary Information

Supplementary Information.

## Data Availability

All data are from the published papers cited.
