# Supplementary material for: Cortical waste clearance in normal and restricted sleep with potential runaway tau buildup in Alzheimer’s disease
Source: Sci Rep. 2022 Aug 12;12:13740. doi: 10.1038/s41598-022-15109-6 (PMC9374764; doi:10.1038/s41598-022-15109-6)
Supplement: Supplementary file 1 — Supplementary Information. [file 41598_2022_15109_MOESM1_ESM.pdf]

Supplementary Information

Cortical Waste Clearance in Normal and Restricted Sleep with Potential Runaway Tau Buildup in Alzheimer's Disease

Tahereh Tekieh, P. A. Robinson, and Svetlana Postnova

S1. Dynamics of One Species

In a fixed arousal state Eqs (6) and (7) in the main text with $\beta_{\sigma i} = 0$ have familiar solutions for saturating exponential rise if $q_{\sigma i} > \chi_{\sigma i}^{(0)} H_i(t)$ and decay otherwise, with

$$H_i(t) = H_i(0) + [H_{\sigma i}^s - H_i(0)] \left[1 - \exp\left(-\chi_{\sigma i}^{(0)} t\right)\right]; \tag{1}$$

a stable fixed point exists at

$$H_{\sigma i}^s = q_{\sigma i}/\chi_{\sigma i}^{(0)}. \tag{2}$$

For $\beta_{\sigma i} \neq 0$ the analytical solution to Eqs (6) and (7) for one chemical species can be obtained by first rewriting these equations as

$$\frac{dt}{dH_i(t)} = \frac{1 + \beta_{\sigma i} H_i(t)}{q_{\sigma i} + [q_{\sigma i}\beta_{\sigma i} - \chi_{\sigma i}^{(0)}] H_i(t)}, \tag{3}$$

which can be integrated to yield

$$H_i(t) - H_i(0) + a \ln\left[\frac{H_i(t) - H_{\sigma i}^s}{H_i(0) - H_{\sigma i}^s}\right] = \frac{\beta_{\sigma i} q_{\sigma i} - \chi_{\sigma i}^{(0)}}{\beta_{\sigma i}}(t - t_0), \tag{4}$$

with the fixed point

$$H_{\sigma i}^s = \frac{q_{\sigma i}}{\chi_{\sigma i}^{(0)} - q_{\sigma i}\beta_{\sigma i}}, \tag{5}$$

and

$$a = \frac{1}{\beta_{\sigma i}} + H_{\sigma i}^s, \tag{6}$$

provided $H_{\sigma i}^s > 0$. Equivalently,

$$e^{[H_i(t) - H_i(0)]} \left[\frac{H_i(t) - H_{\sigma i}^s}{H_i(0) - H_{\sigma i}^s}\right]^a = \exp\left[\frac{\beta_{\sigma i} q_{\sigma i} - \chi_{\sigma i}^{(0)}}{\beta_{\sigma i}}(t - t_0)\right], \tag{7}$$

In general, the transcendental equations (4) and (7) cannot be solved explicitly for $H(t)$. However, if runaway occurs and $H_i(t) \gg H_i(0)$, we find

$$H_i(t) \approx H_i(0) + \frac{\beta_{\sigma i} q_{\sigma i} - \chi_{\sigma i}^{(0)}}{\beta_{\sigma i}}(t - t_0), \tag{8}$$

which reflects the fact that $\chi_{\sigma i}^{(0)} \approx \chi_{\sigma i}^{(0)}/\beta_{\sigma i}$ at large H_i, so there is runaway without eventual saturation, provided

$$q_{\sigma i} > \chi_{\sigma i}^{(0)}/\beta_{\sigma i}. \tag{9}$$

S2. Dynamics of Two Species

If we consider Eqs (6) and (7) in the main text for two species ($i = 1, 2$) and assume that species 1 dominates the dynamics, at least initially, then in a fixed arousal state with $\beta_{\sigma_i} = 0$ the solution of total H is given by Eq. (5) in the main text. When $\beta_{\sigma_i} \neq 0$ Eqs (6) and (7) cannot be solved analytically, but the stable fixed point satisfies

$$H_{\sigma 1}^s = \frac{q_{\sigma 1}\chi_{\sigma 2}^{(0)}}{\Delta}, \tag{10}$$

$$\Delta = \left[\chi_{\sigma 1}^{(0)} - \beta_{\sigma 1} q_{\sigma 1}\right]\left[\chi_{\sigma 2}^{(0)} - \beta_{\sigma 2} q_{\sigma 2}\right] - \beta_{\sigma 1}\beta_{\sigma 2} q_{\sigma 1} q_{\sigma 2}, \tag{11}$$

$$H_{\sigma 2}^s = \frac{q_{\sigma 2}(1 + \beta_{\sigma 1} H_{\sigma 1}^s)}{\chi_{\sigma 2}^{(0)} - \beta_{\sigma 2} q_{\sigma 2}}, \tag{12}$$

so long as runaway does not occur.

Suppose the dynamically dominant species, labeled 1 without loss of generality, runs away and $\beta_{\sigma 1} H_{\sigma 1} \gg \beta_{\sigma 2} H_{\sigma 2}$, then Eq. (8) applies. We term this *overt runaway* because the species that dominates the dynamics runs away so the dynamics undergo immediate significant changes. In this case, the ratio of the two concentrations approaches a limit

$$\gamma = \lim_{t\to\infty} \frac{H_{\sigma 2}(t)}{H_{\sigma 1}(t)}, \tag{13}$$

so the second species' dynamics are slaved to those of the first. The total homeostatic level then obeys

$$\frac{dH_\sigma}{dt} = q_{\sigma 1} + q_{\sigma 2} - \frac{\left(\chi_{\sigma 1}^{(0)} + \gamma\chi_{\sigma 2}^{(0)}\right) H_{\sigma 1}}{1 + (\beta_{\sigma 1} + \gamma\beta_{\sigma 2}) H_{\sigma 1}}. \tag{14}$$

At large $H_{\sigma 1}$ the runaway condition thus becomes

$$q_{\sigma 1} + q_{\sigma 2} > \frac{\chi_{\sigma 1}^{(0)} + \gamma\chi_{\sigma 2}^{(0)}}{\beta_{\sigma 1} + \gamma\beta_{\sigma 2}}. \tag{15}$$

One can calculate the value of γ by considering the dynamics at high concentrations, namely

$$\gamma \approx \frac{dH_{\sigma 2}(t)}{dH_{\sigma 1}(t)}, \tag{16}$$

$$= \frac{(\beta_{\sigma 1} + \gamma\beta_{\sigma 2})q_{\sigma 2} - \gamma\chi_{\sigma 2}^{(0)}}{(\beta_{\sigma 1} + \gamma\beta_{\sigma 2})q_{\sigma 1} - \chi_{\sigma 1}^{(0)}}, \tag{17}$$

which can be solved to give

$$\gamma \approx \frac{1}{2\beta_{\sigma 2}q_{\sigma 1}}\left[b + \sqrt{b^2 + 4\beta_{\sigma 1}\beta_{\sigma 2}q_{\sigma 1}q_{\sigma 2}}\right], \tag{18}$$

$$b = \chi_{\sigma 1}^{(0)} - \chi_{\sigma 2}^{(0)} - \beta_{\sigma 1}q_{\sigma 1} + \beta_{\sigma 2}q_{\sigma 2}. \tag{19}$$
